# Supplementary material for: Empirical methods for controlling false positives and estimating confidence in ChIP-Seq peaks
Source: BMC Bioinformatics. 2008 Dec 5;9:523. doi: 10.1186/1471-2105-9-523 (PMC2628906; doi:10.1186/1471-2105-9-523)
Supplement: Additional File 1 — A variety of html documents from the USeq web site detailing the available applications, their best usage, output file type descriptions, command line menus, etc. [file 1471-2105-9-523-S1.zip › USeqUserGuides/JavaDoc.zip!/JavaDoc/expr/ExpressedGene.html]

ExpressedGene


|  |  |  |
| --- | --- | --- |
| **Overview** \| **Package** \| **Class** \| **Use** \| **Tree** \| **Deprecated** \| **Index** \| **Help** | |  |
| **PREV CLASS**   **NEXT CLASS** | **FRAMES**    **NO FRAMES**     **All Classes** |  |
| SUMMARY: NESTED \| FIELD \| CONSTR \| METHOD | DETAIL: FIELD \| CONSTR \| METHOD |  |


---


## expr Class ExpressedGene

```
java.lang.Object
  expr.ExpressedGene
```

**All Implemented Interfaces:**: java.lang.Comparable

---

``` public class ExpressedGene extends java.lang.Object implements java.lang.Comparable ```

---

**Constructor Summary**

`ExpressedGene(java.lang.String name,
Coordinate coordinates,
float[] values)`


| **Method Summary** | |
| --- | --- |
| `int` | `compareTo(java.lang.Object other)`             Sorts by Coordinate |
| `Coordinate` | `getCoordinates()` |
| `java.lang.String` | `getName()` |
| `RankedFloatArray` | `getRfa()` |
| `float[]` | `getValues()` |
| `void` | `setRfa(RankedFloatArray rfa)` |
| `void` | `setValues(float[] values)` |
| `static ExpressedGene[][]` | `splitByChromosome(ExpressedGene[] exprGenes)`             Splits a ExpressedGene[] by chromosome to ExpressedGene[chrom][lines]. |
| `java.lang.String` | `toString()` |

**Methods inherited from class java.lang.Object**

`equals, getClass, hashCode, notify, notifyAll, wait, wait, wait`

**Constructor Detail**

### ExpressedGene

```
public ExpressedGene(java.lang.String name,
                     Coordinate coordinates,
                     float[] values)
```


**Method Detail**

### toString

```
public java.lang.String toString()
```

:   **Overrides:**: `toString` in class `java.lang.Object`

---


### compareTo

```
public int compareTo(java.lang.Object other)
```

:   Sorts by Coordinate

    :   **Specified by:**: `compareTo` in interface `java.lang.Comparable`

---


### splitByChromosome

```
public static ExpressedGene[][] splitByChromosome(ExpressedGene[] exprGenes)
```

:   Splits a ExpressedGene[] by chromosome to ExpressedGene[chrom][lines].
    Assumes the ExpressedGene[] is sorted by chromosome!

---


### getCoordinates

```
public Coordinate getCoordinates()
```

---


### getName

```
public java.lang.String getName()
```

---


### getValues

```
public float[] getValues()
```

---


### getRfa

```
public RankedFloatArray getRfa()
```

---


### setRfa

```
public void setRfa(RankedFloatArray rfa)
```

---


### setValues

```
public void setValues(float[] values)
```


---


|  |  |  |
| --- | --- | --- |
| **Overview** \| **Package** \| **Class** \| **Use** \| **Tree** \| **Deprecated** \| **Index** \| **Help** | |  |
| **PREV CLASS**   **NEXT CLASS** | **FRAMES**    **NO FRAMES**     **All Classes** |  |
| SUMMARY: NESTED \| FIELD \| CONSTR \| METHOD | DETAIL: FIELD \| CONSTR \| METHOD |  |


---
